# Cytoplasmic expression of LGR5 in pancreatic adenocarcinoma

**DOI:** 10.3389/fphys.2013.00269

**Published:** 2013-09-26

**Authors:** Nobumasa Mizuno, Yasushi Yatabe, Kazuo Hara, Susumu Hijioka, Hiroshi Imaoka, Yasuhiro Shimizu, Shigeru B. H. Ko, Kenji Yamao

**Affiliations:** ^1^Department of Gastroenterology, Aichi Cancer Center HospitalNagoya, Japan; ^2^Department of Pathology and Molecular Diagnostics, Aichi Cancer Center HospitalNagoya, Japan; ^3^Department of Gastroenterological Surgery, Aichi Cancer Center HospitalNagoya, Japan; ^4^Sakaguchi Laboratory, Department of Systems Medicine, Keio University School of MedicineTokyo, Japan

**Keywords:** LGR5, CD133, cancer stem cell, endocrine cell

## Abstract

**Background:** CD133 has been identified as a cancer stem cell marker for pancreatic ductal adenocarcinoma. Although leucine-rich-repeat-containing G-protein-coupled receptor 5 (LGR5), a marker of intestinal stem cells, has been shown to be on a higher level of the stem cell hierarchy than CD133, the expression and function of LGR5 in pancreatic cancer tissue remains unclear. This study investigated tissue expression of LGR5 and CD133 in resected pancreatic cancer tissue.

**Methods:** LGR5 and CD133 expression was immunohistochemically examined in 9 patients with pancreatic ductal adenocarcinoma who underwent resection.

**Results:** LGR5 was expressed in the cytoplasm of pancreatic cancer cells in 4 of 9 cases. CD133 was not detected in cancerous tissue. In non-neoplastic tissue, LGR5 was expressed in the basolateral membrane of a subset of endocrine cells. Conversely, CD133 was expressed in the apical membrane of small duct cells. Co-localization of LGR5 and CD133 was not found in either neoplastic or non-neoplastic tissue. LGR5 expression in pancreatic cancer cells showed no statistically significant correlation with survival after surgery.

**Conclusion:** We have demonstrated that LGR5 is expressed in the cytoplasm of pancreatic adenocarcinoma cells, and the basolateral membrane of a subset of endocrine cells of the human pancreas. Further investigation is required to clarify any prognostic significance of LGR5 expression.

## Introduction

Pancreatic ductal adenocarcinoma (PDA) is a highly aggressive disease usually diagnosed in an advanced stage and for which effective therapies remain lacking. Increasing evidence suggests that stem cells play a decisive role not only in the generation of complex multicellular organisms, but also in the development and progression of tumors (Clevers, 2011). Many tumors have been shown to harbor a subset of distinct cancer cells that bear stem cell characteristics, termed cancer stem cells (CSCs). CSCs are hypothesized to be exclusively responsible for tumor initiation, propagation, and metastasis. In addition, CSCs are thought to be highly resistant to chemo- and radiotherapy.

To date, several CSC markers of pancreatic cancer cells have been identified. A highly tumorigenic CD44+CD24+EpCAM+ cell subpopulation, displaying typical stem cell features, could initiate tumors at low cell numbers, using a xenograft model of immunocompromised mice for human pancreatic cancer cells (Li et al., 2007). CD133 (Hermann et al., 2007), aldehyde dehydrogenase 1a1 (Jimeno et al., 2009), and c-Met (Li et al., 2011) have since been identified as other CSC markers of pancreatic cancer cells.

We have previously demonstrated that corticosteroids induce regeneration of acinar cells in patients with autoimmune pancreatitis (Ko et al., 2010). In that study, we showed that the presence of CD133-positive ductal cells correlate with the regeneration of acinar cells, and thus, play an important role in organ regeneration. These data indicated that CD133 is a good marker for pancreatic stem/progenitor cells.

Clevers and colleagues identified Wnt target gene, leucine-rich-repeat-containing G-protein-coupled receptor 5 (LGR5) as a marker for the intestinal stem cells from which all cellular linages of gastrointestinal epithelium are derived (Barker et al., 2007; Barker and Clevers, 2010). A recent study suggests that LGR5 is on a higher level of the stem cell hierarchy than CD133 (Snippert et al., 2009).

We hypothesized that LGR5 is a stem cell marker of pancreatic cancer cells on a higher level of the stem cell hierarchy than CD133. However, LGR5 expression and its function in pancreatic cancer cells remain unclear. The present study therefore, investigated the tissue expression of LGR5 and CD133 in resected pancreatic cancer tissue.

## Patients and methods

### Patients

Nine of 109 patients with PDA who had undergone pancreatic resection at Aichi Cancer Center Hospital between 2005 and 2010 were included in this pilot study to explore a possible implication of LGR5 for survival. Five patients had a short survival of 1-year or less, and four survived longer than 3 years. The study protocol was approved by our institutional review board. The study was conducted in accordance with the Declaration of Helsinki.

### Immunohistochemistry

Surgically resected tissues were fixed in 10% formalin and embedded in paraffin. Sections were deparaffinized, permeabilized, and used for immunohistochemistry. Antigen retrieval was performed by heating in 0.01 M citrate buffer (pH 6.0) in a microwave. The primary antibodies used for immunohistochemistry included rabbit polyclonal antibody to LGR5 (ab75732; Abcam, Cambridge Science Park, UK) and mouse monoclonal antibody to CD133 (MB9-3G8; Miltenyi Biotec, Germany). Dilutions for all antibodies followed the manufacturer's recommendations. Immunoreactions were intensified using Envision plus reagent (DAKO, Carpinteria, CA). Immunolabeling was visualized using 3,30-diaminobenzidine (DAB) as substrate for horseradish peroxidase. Sections were counterstained with Mayer's hematoxylin. LGR5 and CD133 immunostaining in the neoplastic tissue was compared to immunoreactivity in the non-neoplastic tissue as an internal positive control. When the non-neoplastic tissue was overstained or understained, immunohistochemical staining was repeated to achieve appropriate status.

### Statistical analysis

Disease-free and overall survival was analyzed using the Kaplan-Meier method and log-rank analysis. Hazard ratios were estimated using of a Cox proportional- hazards model. All statistical tests were two-sided, and statistical significance was defined for values of *P* < 0.05. JMP version 9.0.3 software (SAS Institute, Cary, NC) was used for all statistical analyses.

## Results

### Baseline characteristics of resected patients with PDA

Nine patients (4 men, 5 women) were enrolled in this study (Table 1). Median age was 64 years (range, 44–72 years). Seven patients underwent pancreaticoduodenectomy and 2 underwent distal pancreatectomy. Tumor grade according to the World Health Organization (WHO) classification was Grade 1 in two patients and Grade 2 in seven patients. The final stage according to 7th edition of Union for International Cancer Control (UICC) classification was stage IIA in one, IIB in six, and IV in two patients.

**Table 1:** **Baseline characteristics of patients with resected pancreatic adenocarcinoma**.

**Case**	**Sex**	**Age (years)**	**Tumor location**	**Surgery**	**TNM categories**			**Stage**	**Tumor grade**
					***T***	***N***	***M***		
1	M	64	Tail	DP	3	1	1	IV	1
2	F	54	Head	PD	3	1	0	IIB	1
3	M	72	Tail	DP	3	1	0	IIB	2
4	F	56	Head	PD	3	1	0	IIB	2
5	M	44	Head	PD	3	1	0	IIB	2
6	M	71	Head	PD	3	1	0	IIB	2
7	F	56	Head	PD	3	0	0	IIA	2
8	F	67	Head	PD	3	1	1	IV	2
9	F	65	Head	PD	3	1	0	IIB	2

PD, pancreaticoduodenectomy; DP, distal pancreatectomy.

### Expression of CD133 in pancreatic cancer tissue

**Figure 1:**
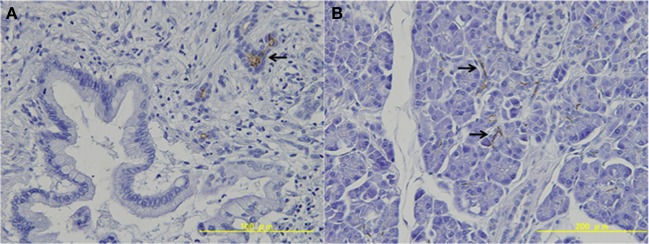
**Expression of CD133 in pancreatic tissue. (A)** Pancreatic cancer cells were negative for CD133. **(B)** CD133 was expressed at the apical membrane of the small pancreatic duct cells in the non-neoplastic tissue (arrows).

All pancreatic cancer cells were negative for CD133 (Table 2, Figure 1A). Conversely, CD133 was expressed at the apical membrane of small pancreatic duct cells in the non-neoplastic tissue around the cancer tissue (Table 2, Figure 1B).

**Table 2:** **Expression of LGR5 and CD133 in pancreatic tissue and survival time**.

**Case**	**LGR5**		**CD133**		**Survival time**			
	**Cancer cells**	**Non-cancer cells**	**Cancer cells**	**Non-cancer cells**	**OS (days)**	**Censored**	**DFS (days)**	**Censored**
1	+	−	−	−	306	no	221	no
2	−	−	−	−	111	no	36	no
3	+	+	−	−	2081	yes	2081	yes
4	+	+	−	+	247	no	201	no
5	−	+	−	+	1283	no	552	no
6	+	−	−	−	2061	yes	2061	yes
7	−	+	−	+	2081	yes	1522	no
8	−	+	−	−	292	no	163	no
9	−	+	−	+	315	no	102	no

OS, overall survival; DFS, disease free survival.

### Expression of LGR5 in pancreatic cancer tissue

**Figure 2:**
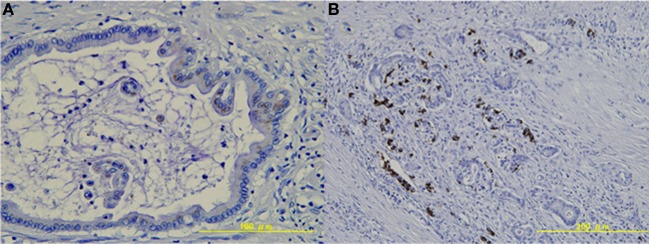
**Expression of LGR5 in pancreatic tissue. (A)** LGR5 was weakly positive in the cytoplasm of pancreatic cancer cells. **(B)** LGR5 was strongly positive in the basolateral membrane of the remaining endocrine cells in non-neoplastic tissue.

Next, we investigated expression of LGR5 in resected pancreatic cancer tissues. In 4 of the 9 cases, LGR5 was weakly positive in the cytoplasm of pancreatic cancer cells (Table 2, Figure 2A). LGR5 was strongly positive in the basolateral membrane of a subset of remaining endocrine cells in non-neoplastic tissue surrounding the pancreatic cancer tissue (Table 2, Figure 2B). On the other hand, LGR5 was negative in the apical membrane of the remaining small pancreatic duct cells that were positive for CD133 (Figure 2B).

### Correlation of LGR5 and prognosis of pancreatic cancer

We investigated correlations between LGR5 expression in pancreatic cancer cells and survival after surgery. Median overall survival has not been reached in the LGR5+ group and 10.4 months in LGR5− group [hazard ratio (HR), 0.52; 95% confidence interval (CI), 0.08–2.99; *P* = 0.52]. Median disease-free survival has not been reached in the LGR5+ group and 5.4 months in LGR5− group (HR, 0.13; 95% CI, 0.04–1.42; *P* = 0.13) (Table 3).

**Table 3:** **Correlation between LGR5 expression and survival**.

	**LGR5 expression**	
	**(−) *n* = 5**	**(+) *n* = 4**
Median OS	10.4	not reached
HR (95% CI)	0.52 (0.08 − 2.99, *P* = 0.52)	
Median DFS	5.4	not reached
HR (95% CI)	0.13 (0.04 − 1.42, *P* = 0.13)	

OS, overall survival; DFS, disease-free survival; HR, hazard ratio; CI, confidence interval.

## Discussion

Central to the CSC concept is the observation that not all cells in tumors are equal. The CSC concept postulates that, similar to the growth of normal proliferative tissue such as bone marrow, skin or intestinal epithelium, the growth of tumors is fueled by a limited number of dedicated stem cells that are capable of self-renewal (Clevers, 2011). This stem cell hypothesis has recently been explored in PDA (Hermann et al., 2007; Li et al., 2007, 2011; Jimeno et al., 2009).

In the present study, CD133 was expressed at the apical membrane of small pancreatic duct cells in the non-neoplastic tissue around the cancer tissue. These results are consistent with our previous data. Hermann et al. reported that human pancreatic cancer tissue contains CSCs defined by CD133 expression that are exclusively tumorigenic and highly resistant to standard chemotherapy (Hermann et al., 2007). They showed that in the invasive front of pancreatic tumors, a distinct subpopulation of CD133+CXCR4+ CSCs determined the metastatic phenotype of the individual tumor. However, pancreatic cancer cells were negative for CD133 in our study. One possible explanation for this discrepancy is the sensitivity of an anti-CD133 antibody. The results of immunohistochemical analysis can vary in frequency from antibody to antibody (Sauter et al., 2009). We used clone MB9-3G8 (Miltenyi Biotec, Germany), which is widely used and recognized as reliable antibody against CD133 (O'Brien et al., 2007; Ricci-Vitiani et al., 2007). In our previous study, we used the same antibody and showed clear immunostaining in the pancreas tissue (Ko et al., 2010). The present results are consistent with our previous study. Another explanation could be the condition of formalin-fixed tissues, because immunohistochemical positivity depends on the methods and times of tissue fixation (Sauter et al., 2009).

Stem cells of the mouse small intestine, colon, and stomach can be identified by the specific expression of LGR5, a G protein-coupled receptor of unknown function. LGR5+ intestinal stem cells are long-lived, proliferating continuously and generating all the cell types present in the gut. The onset of intestinal tumorigenesis is driven in most cases by activation of Wnt signal pathways. Mouse LGR5- positive cells give rise to intestinal tumors with higher efficiency than other intestinal cell populations upon mutational activation of the Wnt pathway (Barker et al., 2009). Therefore, LGR5- positive cells are thought to represent candidates for CSCs of colorectal cancer. Snippert et al. reported that CD133 marks intestinal stem cells, as well as transit-amplifying progenitors (Snippert et al., 2009). The expression of LGR5 in pancreatic tissue has not been investigated. The present study showed that LGR5was expressed in the cytoplasm of some pancreatic cancer cells. LGR5 was also positive in the basolateral membrane of the remaining endocrine cells surrounding the pancreatic cancer tissue. Immunoreactivity of LGR5 in endocrine cells was stronger than that in pancreatic cancer cells. On the other hand, LGR5 did not co-localize with CD133 in pancreatic cancer tissue.

Simon et al. recently studied the prevalence, histoanatomical distribution and tumor biological significance of LGR5 in tumors of the human gastrointestinal tract (Simon et al., 2012). That study found that LGR5 expression was positive in all 17 cases (100%) with PDA tissue and in 12 of 17 cases (71%) in non-neoplastic tissue. Localization of LGR5 expression was observed as mainly cytoplasmic, but a sporadic membrane or core membrane accentuated expression occurred. Localization of LGR5 in pancreatic cancer cells is consistent with our study. However, the positive rate for LGR5 was higher than in our result (4 of 9, 44%). One possible explanation for this discrepancy is the use of different antibodies against anti-LGR5. They used anti-LGR5− antibody generated by themselves, while we used a commercial polyclonal antibody against LGR5. We have tested reliability of several antibodies against LGR5 prior to this study. Therefore, we used ab75732 (Abcam, Cambridge Science Park, UK) as an antibody against LGR5 in the present study. Immunoreactivity of LGR5 for endocrine cells was consistent with our previous study (Ko et al., 2013). Another possible explanation is the differences in ethnic background. Although LGR5expression has not been studied across ethnic groups, proportions of other colorectal CSC markers vary according to ethnic background (Leavell et al., 2012).

The CSC hypothesis predicts that stem cells are responsible for tumor initiation and preferentially drive tumor growth. Patients with LGR5+ colorectal cancer and gastric cancer reportedly show shorter survival than patients with LGR5− (Merlos-Suarez et al., 2011; Simon et al., 2012). On the contrary, using mouse models of glioma cells, Barrett et al. described that high expression of ld1 identifies tumor cells with high self-renewal capacity, while low ld1 expression identifies tumor cells with proliferative potential but low self-renewal capacity (Barrett et al., 2012). Their results argue against stringent interpretation of the CSC hypothesis. In our study, hazard ratios for OS and DFS with LGR5- positive were 0.52 and 0.13, respectively, however, there were no statistically significant differences. Moreover, stage distribution varied among LGR5- positive (stage IIB 3, IV 1) and negative (stage IIA 1, IIB 3, IV 1) groups, because cases were not matched for stage. Inadequate statistical power with small sample size (*n* = 9) and an absence of case-control study design are limitations of this study. Further studies with adequate statistical power and stage-matched cases are needed to verify the prognostic implications of LGR5 in pancreatic cancer, if any.

Wnt signaling plays an important role in the activation of the mammalian target of rapamycin (mTOR) pathway to stimulate intestinal polyp formation (Fujishita et al., 2008). Activation of the mTOR pathway has also been implicated in the proliferation of pancreatic neuroendocrine tumors. A recent study suggests that Wnt/β-catenin signaling contributes to the pathogenesis and growth of neuroendocrine tumors (Kim et al., 2013). LGR5 was expressed in the basolateral membrane of a subset of endocrine cells of the pancreas in our study. Although LGR5 function in endocrine cells and neuroendocrine tumor cells remains unclear, LGR5 might represent a putative stem cell marker of matured neuroendocrine cells and neuroendocrine tumor of the pancreas (Ko et al., 2013). Recent study suggests that LGR5 is expressed in the remaining islets and in ductal cancer cells in cancerous pancreas, therefore, pancreatic islets beta cells contain cells-of-origin of PDA that express their unique markers in the PDA tumor cells (Amsterdam et al., 2013).

In conclusion, we have demonstrated that LGR5 is expressed in the cytoplasm of pancreatic cancer cells and the basolateral membrane of endocrine cells of the pancreas in patients with PDA. Further investigations are required to clarify the biological functions of LGR5 and its possible application as a stem cell marker for pancreatic exocrine and endocrine tumors.

### Conflict of interest statement

The authors declare that the research was conducted in the absence of any commercial or financial relationships that could be construed as a potential conflict of interest.
